# Analysis of Internal and External Microorganism Community of Wild Cicada Flowers and Identification of the Predominant *Cordyceps cicadae* Fungus

**DOI:** 10.3389/fmicb.2021.752791

**Published:** 2021-11-25

**Authors:** Ailin Huang, Tao Wu, Xiuyun Wu, Biao Zhang, Yuanyuan Shen, Suying Wang, Wenjun Song, Haihua Ruan

**Affiliations:** Tianjin Key Laboratory of Food Science and Biotechnology, College of Biotechnology and Food Science, Tianjin University of Commerce, Tianjin, China

**Keywords:** adenosine, artificial cultivation, microbial community, *N*^6^-(2-hydroxyethyl)-adenosine, soil physicochemical properties

## Abstract

The parasitoid fungus *Cordyceps cicadae*, whose fruiting bodies are known in China as “chan hua,” literally “cicada flower,” has been used as a traditional Chinese medicinal ingredient for centuries. However, systematic disclosure of the vital factors responsible for the formation of wild cicada flower is limited. Here, we determined the physicochemical properties of soil and simultaneously analyzed the diversities and the structures of microbial community inhabiting the coremia, sclerotia, and soil around wild cicada flowers through high-throughput sequencing. Our results indicated that cicada flower more preferentially occurred in acidic soil (pH 5.9) with abundant moisture content (MC), total nitrogen (TN), and organic matter (OM). The dominant fungal genera in soil mainly included *Isaria*, f__Clavariaceae_Unclassified, *Umbelopsis*, f__Chaetomiaceae_Unclassified, *Mortierella*, f__Sordariaceae_Unclassified, and *Arcopilus*. Among them, *C. cicadae* was the only fungus that was massively detected in both the coremia and sclerotia with abundance of 83.5 and 53.6%, respectively. Based on this, a *C. cicadae* strain named AH10-4 with excellent adenosine- and *N*^6^-(2-hydroxyethyl)-adenosine (HEA)-producing capability was successfully isolated. However, to the aspect of bacteria, *Burkholderia–Caballeronia–Paraburkholderia*, *Bacillus*, *Acidibacter*, f__Xanthobacteraceae_Unclassified, and *Candidatus_Solibacter* were the dominant genera in soil. *Pedobacter*, f__Enterobacteriaceae_Unclassified, *Pandoraea*, *Achromobacter*, *Stenotrophomonas*, *Burkholderia–Caballeronia–Paraburkholderia*, and *Chitinophaga* were the dominant genera in the coremia and sclerotia. Notably, *Burkholderia–Caballeronia–Paraburkholderia* was the shared bacteria among them with high abundance of 3.1, 11.4, and 5.2% in the sclerotia, coremia, and soil, respectively. However, the possible role of these bacteria to the occurrence of cicada flower has been unclear to our knowledge. By analyzing the correlation between physicochemical properties and microbial community of soil, we found that MC, Fe, and Zn were significantly negatively correlated with soil *Isaria* and that Cu was significantly negatively correlated with most dominant soil bacterial genera. But Mg was significantly positively correlated with most dominant taxa. This study provides new insight into the formation mechanisms of cicada flower and may contribute to the large-scale cultivation of cicada flowers.

## Graphical Abstract

Brief strategy for wild cicada flowers internal and external microorganism community analysis and its applications.

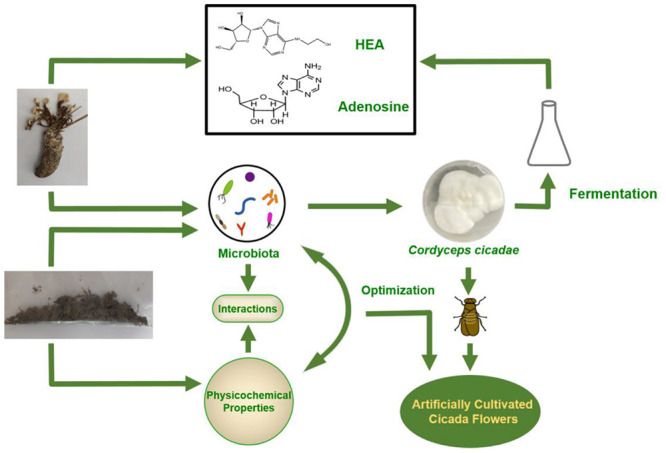

## Introduction

Chan hua, literally “cicada flowers” (Dong et al., 2015), are the fruiting bodies of the fungus *Cordyceps cicadae* (syn. *Isaria cicadae* and *Paecilomyces cicadae*) (Chunyu et al., 2019), which is a parasitic complex fungus that grows inside cicada nymphs and larvae and forms fruiting bodies on the surface of the insect carcass (Paterson, 2008). The medicinal value of cicada flower was firstly recorded in the Chinese medical text “Lei Gong Pao Zhi Lun” about 1,600 years ago (Liu et al., 2019). As a valuable traditional Chinese medicinal tonic, cicada flower has received significant attention from the researchers; and a number of studies have shown that cicada flower has various health effects, such as treating fever, improving vision, protecting renal function, and treating infantile convulsions, dizziness, and palpitations (Lu et al., 2015). In addition, modern pharmacological studies have demonstrated that cicada flower possesses antitumor (Xie et al., 2019), anti-fatigue (Liu et al., 2019), anti-inflammatory (Zheng et al., 2018), immunomodulatory, analgesic (Kim et al., 2011), sedative (Weng et al., 2002), and renoprotective (Zhu et al., 2014) effects.

In this paper, to avoid misunderstanding, we use cicada flower for the fungus-larva symbiote and *C. cicadae* or *I. cicadae* for the fungus. A series of bioactive substances have been isolated and identified from cicada flower to date. Among them, nucleosides, such as adenosine, guanosine, and *N*^6^-(2-hydroxyethyl)-adenosine (HEA), represent the most abundant bioactive components of *Cordyceps* (Meng et al., 2019). Known to all, cicada flower grows in a specific environment, where soil properties and the microbial community may play crucial roles in the formation of its fruiting bodies.

As a traditional Chinese medicinal ingredient, cicada flower has attracted increasing attention in the scientific community due to its active ingredients. However, the yield of wild cicada flower is extremely limited due to its complex seasonal life cycle. The appearance of fruiting bodies is the culmination of the infection, colonization, and growth of an entomogenous fungus (*C. cicadae*) of cicada nymphs and larvae in the soil. Previous studies revealed that the growth of fungi is dependent on physical, chemical, and microbial properties of the soil (de Boer et al., 2003; Wu et al., 2008; Bonanomi et al., 2013). Moreover, the soil microbial community and activity are directly or indirectly influenced by the physicochemical characteristics (Wang et al., 2018; Xu et al., 2020); and conversely, the microbes in soil play an important role in biogeochemical cycling and are therefore indicators of soil quality (Ghosh et al., 2004; Xia Z. et al., 2016). Based on this, the soil microenvironment is closely related to the survival of *C. cicadae* and the occurrence of cicada flower. The soil physicochemical properties and the internal and external microbial communities therefore critically influence the formation of the medicinal fruiting bodies in the wild.

Although high-throughput sequencing has been used to explore the microbial community associated with *Ophiocordyceps sinensis* (Zhang et al., 2010; Xia F. et al., 2016; Shao et al., 2019; Xia et al., 2019), only a few papers investigated the bacterial and fungal communities inhabiting cicada flower. Previous researches have revealed that cicada flowers enrich abundant microbial communities (Chunyu et al., 2019; Qu et al., 2019; Mou et al., 2020; Zeng et al., 2021), and the microbial community composition varies with the geographic area (Chunyu et al., 2019), and the endophytic bacteria in cicada flowers can influence the metabolite production (Qu et al., 2019). These results suggested that wild cicada flower populations are ecologically linked to the surrounding soil through microorganisms, which may impact cicada flower growth and production of metabolites.

Recent studies have shown the potential for dietary/medicinal application of cicada flower, but the increasing attention has also sparked unsustainable harvesting from the wild, coupled with the deteriorating ecological environment, leading to natural cicada flower resources becoming increasingly tense. Therefore, in order to meet the enormous demand in the market, artificial cultivation of cicada flowers has become a focus of research. Moreover, cultivating cicada flowers in natural habitat soil was proved to be feasible (Zeng et al., 2021), but there are no systematic studies focused on the growth environment of cicada flower. Therefore, it is of great significance to explore the growth mechanism and growth environment of cicada flower. Besides, isolation and breeding of excellent *C. cicadae* strains are a key step in artificially cultivating cicada flower in the future. Thus, to increase the production of cicada flowers in artificial cultivation, it is essential to obtain the *C. cicadae* strains with high production of active substances and to explore the vital environmental factors that affect the production of cicada flowers.

In this study, we aimed to systematically explore the physicochemical characteristics and the internal and external microbial communities of cicada flower to with an emphasis on their influence on its occurrence and isolate and identify the predominant *C. cicadae* fungus for the next stage of research. To achieve this, we not only analyzed the physical and chemical properties of the surrounding soil that have an important impact on the occurrence of cicada flower but also explored the internal and external microbial communities of the fruiting bodies using high-throughput sequencing. Additionally, we isolated the key fungal strain belonging to the dominant species in the fruiting body, and the metabolites of the isolated *C. cicadae* strain were evaluated in shake flask culture. These findings lay a foundation for further research into the diversity of natural associated microorganisms inhabiting the cicada flower and provide new ideas for its large-scale cultivation.

## Materials and Methods

### Sample Preparation

Wild cicada flowers in China are mainly distributed in the four provinces Anhui, Jiangsu, Sichuan, and Zhejiang. In this study, wild cicada flower and soil were collected from Anji County in Zhejiang Province, China. Our pre-survey revealed that the average density of natural cicada flower in Anji is 0.5 fruiting bodies per square meter, with the highest density being 5 fruiting bodies per square meter. Field investigations revealed that the peaks of wild cicada flowers usually occurred annually from June to August.

Wild cicada flower and soil samples were sampled by the diagonal line five-point method in mid-July 2020. During the sampling, weeds on the ground were removed first, after which the soil was cut into V-shaped pits by using a plastic shovel, and soil slices 5–10 cm deep and 10 cm wide were collected. The soil samples were divided into the cicada flower group (collected from the soil around the fruiting bodies) and the null cicada flower group (collected from a locality where no cicada flower was observed for 10 years). The samples were stored in sterile plastic bags and sent to the laboratory in an ice box. According to the morphological and anatomical characteristics, the wild cicada flowers were divided into two parts: (1) coremium, the aboveground part; and (2) sclerotium, the underground part (a cicada pupa filled with the mycelia of *C. cicadae*) (Chunyu et al., 2019). The processing and storage method of the collected wild cicada flowers were according to Xia et al. (2015). The fresh samples were stored at −20°C until DNA was extracted. Each soil sample was divided into two parts, one of which was used for DNA extraction and the other for analysis of soil physicochemical properties.

### Determination of Soil Physicochemical Properties

Thirteen physicochemical properties of soil were analyzed, including pH, moisture content (MC), total phosphorus (TP), total nitrogen (TN), available phosphorus (AP), available potassium (AK), organic matter (OM), and magnesium (Mg), aluminum (Al), copper (Cu), iron (Fe), manganese (Mn), and zinc (Zn).

The soil pH (1:2.5, soil:water) was assayed potentiometrically according to HJ 962-2018, China. The MC was determined using a gravimetric method by weighing samples before and after oven-drying at 105°C for 24 h. The determination of TP and AP content was according to LY/T 1232-2015, China. TP content was assessed using the NaOH melting method, which is the Mo–Sb anticolorimetric method; AP was determined using the sodium bicarbonate method. According to LY/T 1228-2015, China, TN was determined using the Kjeldahl method. The determination of AK was according to LY/T 1234-2015, China; AK was determined by atomic adsorption spectrophotometry after the soil was extracted with 1 mol/L of ammonium acetate, pH 7. OM in the soil was assayed using the potassium dichromate titrimetric method in an electric sand bath according to LY/T 1237-1999, China. Mg was measured by atomic absorption spectrophotometer after digested with perchloric acid and hydrofluoric acid according to NY/T 296-1995, China. The determination of Mn, Cu, and Zn was according to HJ 803-2016, China. Mn, Cu, and Zn were detected by an inductively coupled plasma mass spectrometer after the soil was digested with HCl–HNO_3_ in a microwave digestion instrument. The determination of Fe and Al was according to LY/T 1253-1999, China. Fe was determined using the phenanthroline spectrophotometric method, and Al was determined using KF replacement–EDTA volumetric method.

### Extraction of Genomic DNA, PCR Amplification, and Sequencing

Before DNA extraction, each wild cicada flower sample was ground into a powder in liquid nitrogen. Total genome DNA from soil and wild cicada flower sample was extracted using the HiPure Soil DNA Kit B according to the manufacturer’s protocol. The DNA concentration was monitored using a Qubit3.0 Fluorometer. The V3 and V4 hypervariable regions of the prokaryotic 16S rRNA gene were amplified using the forward primer “CCTACGGRRBGCASCAGKVRVGAAT” and reverse primer “GGACTACNVGGGTWTCTAATCC.” The ITS2 region was amplified using the forward primer “GTGAATCA TCGARTC” and reverse primer “TCCTCCGCTTATTGAT.”

PCR for bacterial-specific fragments was performed using 3 min of denaturation at 94°C; 24 cycles of 5 s at 94°C, 90 s for annealing at 57°C and 10 s for elongation at 72°C; and a final extension at 72°C for 5 min. For fungal-specific fragments, the PCR procedure included 5 min of denaturation at 94°C; 25 cycles of 30 s at 94°C, 30 s for annealing at 57°C, and 30 s for elongation at 72°C; and a final extension at 72°C for 5 min. The resulting PCR products were evaluated by 1.5% agarose gel electrophoresis. The PCR reaction mixture (25 μl) contained 2.5 μl of TransStart Buffer, 2 μl of dNTPs, 1 μl of each primer, and 20 ng of template DNA.

Next-generation sequencing (NGS) library generation and Illumina MiSeq sequencing were conducted by GENEWIZ, Inc. (Tianjin, China). The concentrations of DNA libraries were validated using a Qubit 3.0 Fluorometer. To normalize the library to 10 nM, DNA libraries were multiplexed and loaded onto an Illumina MiSeq instrument according to the manufacturer’s instructions (Illumina, San Diego, CA, United States). Sequencing was performed in PE250/300 paired-end mod; image analysis and base calling were conducted using the MiSeq Control Software (MCS) embedded in the MiSeq instrument.

### Phylogenetic Analyses

The QIIME data analysis package was used for 16S rRNA and ITS rRNA data analysis. The forward and reverse reads were joined by Quantitative Insights Into Microbial Ecology (QIIME) software (Caporaso et al., 2010) and assigned to samples based on barcode and truncated by cutting off the barcode and primer sequence. Quality filtering on joined sequences was performed, and sequences that did not fulfill the following criteria were discarded: sequence length < 200 bp, no ambiguous bases, mean quality score ≥ 20. Then the sequences were compared with the reference database [Ribosomal Database Program (RDP) Gold database] using UCHIME algorithm to detect chimeric sequence, and then the chimeric sequences were removed. The effective sequences were used in the final analysis. Sequences were grouped into operational taxonomic units (OTUs) using the clustering program VSEARCH (1.9.6) against the Silva 132 database and the UNITE ITS database^1^ pre-clustered at 97% sequence identity. The RDP classifier uses the Silva (Pruesse et al., 2007) 132 and the UNITE (Koljalg et al., 2005) ITS database, which has taxonomic categories predicted to the species level.

### Isolation and Identification of *Cordyceps cicadae* Strain

The coremium was placed into a 15-ml centrifuge tube with 6 ml of 0.9% NaCl solution and shaken to liberate the fungal spores. Aliquots comprising 200 μl of serial dilutions of each sample (10^–1^, 10^–2^, 10^–3^, and 10^–4^) were spread on plates containing rose bengal agar (RBA), and the dishes were placed in a 26°C incubator for culture. When the colony diameter reached 2–5 mm, a small amount of mycelium was removed and purified 2–4 times on the separation medium. After it was confirmed that there were no other microorganisms, the strains were transferred to potato-dextrose agar (PDA) slants, incubated at 26°C for 7 days, and then stored at 4°C.

The mycelia of the fungal strain growing on PDA were collected, and the fungal genomic DNA was extracted using the E.Z.N.A.^®^ Fungal DNA Mini Kit. The ITS rRNA gene was amplified by PCR using the universal primers ITS1F (5′-TCCGTAGGTGAACCTGCGG-3′) and ITS4R (5′-TCCTCCGCTTATTGATATGC-3′). The amplified products were separated by 1% agarose gel electrophoresis and sequenced by GENEWIZ, Inc. (Tianjin, China). The sequences were searched against the National Center for Biotechnology Information (NCBI) database using the Basic Local Alignment Search Tool (BLAST), and the selected sequences were aligned using CLUSTALW. Molecular Evolutionary Genetics Analysis (MEGA-X) software was used to construct the phylogenetic tree based on the neighbor-joining algorithm.

### Submerged Fermentation of *Cordyceps cicadae* and Quantitative Analysis of Nucleosides

The activated strain was grown on PDA medium at 26°C for 7 days. Spores of *C. cicadae* were then used to inoculate the fermentation medium and cultured at 26°C and 140 rpm for 3 days. The resulting seed culture was used to inoculate 100 ml of medium (inoculum amount: 5%) and cultured at 26°C and 140 rpm for 5 days. The composition of the seed culture/fermentation medium was extracted by a previous method with some modifications and contained (per liter) the following: 20 g of glucose, 10 g of peptone, 10 g of yeast extract, 2.0 g of KH_2_PO_4_, 0.5 g of MgSO_4_, and 0.5 g of ZnCl_2_ (Chunyu et al., 2019).

The mycelia were harvested by centrifugation at 5,900 × *g* for 30 min and then oven-dried at 60°C. The dried mycelia, coremia, and sclerotia were ground into a powder, and 0.3 g of the powder was ultrasonically extracted with ultrapure water for 90 min. The resulting extract was cleared from debris by centrifugation at 5,900 × *g* for 15 min. The supernatant was filtered through a 0.22-μm pore-size Minisart^®^ filter for later use.

Since nucleosides are water-soluble, their presence in the water extracts of wild cicada flowers and artificially cultured *C. cicadae* mycelia was analyzed by high-performance liquid chromatography (HPLC) on an Agilent ZORBAX Eclipse XDB-C18 column (150 mm × 4.6 mm × 5 μm). The mobile phase consisted of 15% methanol in ultrapure water at a flow rate of 1.0 ml/min. The injection volume was 10 μl, and the detection wavelength was set at 260 nm.

### Statistical Analysis

The statistical significance of differences among different groups was determined using the paired-samples *t*-test and one-way ANOVA for repeated measures in SPSS software version 23.0 (IBM Corp., Armonk, NY, United States). Differences at the level of *p* < 0.05 were considered statistically significant.

## Results

### Soil Physicochemical Properties

The wild cicada flower mainly grows in bamboo, broad-leaved and evergreen broad-leaved forests, and mixed coniferous and broad-leaved forests. The wild cicada flower we acquired from Anji County in Zhejiang Province was characterized by high quality and yield due to the warm climate, loose soil, and high MC.

In order to disclose the effects of various elements from the soil on the formation of wild cicada flower, the physicochemical properties of soil were determined. As shown in Table 1, compared with the control soil group without cicada flower growth, the cicada flower group exhibited significantly higher levels of MC, TN, and OM, as well as significantly lower levels of pH, TP, AP, AK, Mg, Fe, Mn, Cu, and Zn (*p* < 0.05).

**TABLE 1:** Physicochemical properties of soil samples.

**Physicochemical property**	**Cicada flower group**	**Control group**
pH	5.917 ± 0.101*	7.963 ± 0.120
MC (%)	45.167 ± 5.052*	22.767 ± 1.528
TN (g/kg)	3.953 ± 0.295*	1.373 ± 0.108
TP (g/kg)	0.336 ± 0.023*	0.768 ± 0.093
OM (g/kg)	89.768 ± 7.382*	32.533 ± 4.477
AP (mg/kg)	3.043 ± 0.156*	28 ± 3.503
AK (mg/kg)	183 ± 19*	548 ± 64.954
Mn (mg/kg)	317.667 ± 45.015*	656.333 ± 43.294
Cu (mg/kg)	7.8331.021*	24.1670.814
Zn (mg/kg)	76.667 ± 8.327*	101.667 ± 3.215
Mg (g/kg)	2.867 ± 0.115*	15.333 ± 0.451
Al (g/kg)	75.533 ± 10.041	55.8 ± 10.137
Fe (g/kg)	26.5 ± 0.819*	36.133 ± 0.306

*p < 0.05 compared with the control group without cicada flower.

*MC, moisture content; TN, total nitrogen; TP, total phosphorus; AP, available phosphorus; AK, available potassium; OM, organic matter.*

### Microbial Diversities

We performed high-throughput rDNA sequencing of soil and different parts of wild cicada flower, including the aboveground part (coremium) and the underground part (sclerotium), to clarify the external and internal microbial communities of cicada flower and to explore the possible interactions between them. In order to explore the diversities of microorganisms, first, alpha-diversity estimators were used to reveal the richness and diversity of species in the community of the coremia, sclerotia, and soil (Table 2). Then, principal component analysis (PCA) plots demonstrated the marked differences in the composition of the coremia, sclerotia, and soil.

**TABLE 2:** Alpha-diversity indices of bacterial and fungal communities in different samples.

	**Sample**	**ACE**	**Chao1**	**Shannon**	**Simpson**	**Good’s_coverage**
	Soil	185.010 ± 9.794	185.889 ± 11.461	6.007 ± 0.575	0.968 ± 0.017	1
Bacterial	Coremia	129.908 ± 27.362*	114.833 ± 10.322*	3.524 ± 0.123*	0.848 ± 0.022*	0.999 ± 0.001
	Sclerotia	105.647 ± 12.287*	98.866 ± 10.996*	3.125 ± 0.287*	0.810 ± 0.038*	1
	Soil	261.713 ± 15.921	260.371 ± 15.719	4.1490.463	0.867 ± 0.043	1
Fungal	Coremia	115.403 ± 16.573*	123.678 ± 15.470*	1.786 ± 1.238*	0.502 ± 0.368	1
	Sclerotia	53.351 ± 31.416^*,#^	53.983 ± 37.741^*,#^	0.413 ± 0.651*	0.177 ± 0.297*	1

*p < 0.05 compared with soil.

*^#^p < 0.05 compared with coremia.*

**FIGURE 1:**
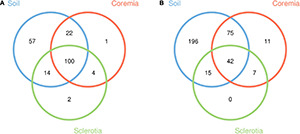
Venn diagrams depicting the unique and shared operational taxonomic units (OTUs) from **(A)** the bacterial and **(B)** the fungal community among the soil, coremia, and sclerotia samples.

Good’s coverage of samples (>0.99 for all samples) and the rarefaction curves (Supplementary Figure 1) indicated that the majority of microbes were represented in the data and that the sampling depth of sequence dataset in the current study was adequate for our analyses. After 72,875 and 55,554 chimeric sequences were filtered, a total of 746,944 high-quality 16S rDNA sequences and 1,196,194 high-quality ITS rDNA sequences were obtained. With a 97% identity threshold, these high-quality sequences were clustered into 200 bacterial and 346 fungal OTUs. The shared and unique OTUs among the soil, coremia, and sclerotia (Figure 1) were depicted using Venn diagrams drawn by the R software based on OTU. Our results indicated that 100 bacterial and 42 fungal OTUs are shared among the soil, coremia, and sclerotia as shown in Figure 1. Aside from that, 57 bacterial OTUs were only found in the soil, while only 1 and 2 OTUs were exclusive to the coremia and sclerotia, respectively. Among fungi, 196 OTUs were only found in the soil, and 11 were only found in the coremia. By contrast, no OTUs were exclusively found in the sclerotia. The detailed taxonomic information of the OTUs is listed in Supplementary Tables 1–4. In summary, the number of total and unique fungal OTUs in the soil was higher than in the coremia or sclerotia.

In order to reveal the richness and diversity of species in the communities among the soil, coremia, and sclerotia, alpha diversity was analyzed using four indices, i.e., ACE, Chao1, Shannon, and Simpson, and was calculated using QIIME. For bacteria, the richness (represented by Chao1 and ACE) and diversity (represented by Shannon and Simpson indices) of communities in the soil was significantly higher than those of the communities in the coremia and sclerotia (*p* < 0.05). However, there was no significant difference between the coremia and sclerotia (*p* > 0.05). For fungi, the richness and diversity of communities in the soil were also significantly higher than those in the coremia and sclerotia (*p* < 0.05), but the fungal richness of the coremia was significantly higher than that of the sclerotia (*p* < 0.05), and there was no significant difference in fungal diversity between the coremia and sclerotia (*p* > 0.05). In conclusion, the richness and diversity of bacterial communities in the coremia and sclerotia were very similar, but the richness of fungal communities in the coremia was significantly higher than in the sclerotia. Notably, *C. cicadae* was dominant in the sclerotia, which may explain their low fungal diversity and richness.

**FIGURE 2:**
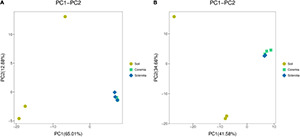
Principal component analysis (PCA) of the **(A)** bacterial and **(B)** fungal communities in the samples of soil, coremia, and sclerotia.

To infer differences of microbial composition between samples, Euclidean distances were calculated for all samples and analyzed in R using the “vegan” package. The results were visualized using PCA. Our results showed that the bacterial (Figure 2A) and fungal communities (Figure 2B) in the soil samples were different from those in the coremia and sclerotia of cicada flower. However, the microbial community in the coremia samples appeared more similar to that of sclerotia samples.

In conclusion, the external microbial environment of cicada flower was richer and more diverse than the internal microbial environment, possibly following the reason that a portion of the microbes was unable to colonize in the internal environment of cicada flowers, which might be inhibited or killed by the fungus during its invasion into cicada nymphs and larvae. The similar microbial compositions of the coremia and sclerotia indicated that some bacteria from the sclerotia might be brought into the coremia during the growth of mycelia within *C. cicadae*, which was consistent with earlier findings that fungal mycelia can promote the short- or long-distance migration of bacteria in the soil (Yang and van Elsas, 2018).

### Fungal and Bacterial Community Structure

**FIGURE 3:**
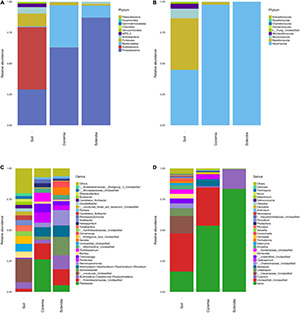
Structure of the endogenous fungal community of cicada flower and its microhabitat samples. **(A)** Endogenous bacterial community at the phylum level. **(B)** Endogenous fungal community at the phylum level. **(C)** Endogenous bacterial community at the genus level. **(D)** Endogenous fungal community at the genus level.

The microbial composition of different samples was analyzed at the phylum and genus levels. Figure 3 intuitively illustrates the top 30 phyla and top 30 genera of the bacterial and fungal communities. The relative compositions of bacterial and fungal communities at the class, order, and family levels are presented in Supplementary Figures 2–7, respectively. The dominant phyla or genera were defined as the phyla or genera whose relative abundance accounted for more than 1% in the soil, coremia, or sclerotia. At the phylum level, the structure of the bacterial community displayed the same patterns between the coremia and sclerotia (Figure 3A). Proteobacteria, Bacteroidetes, and Firmicutes were the dominant phyla in both the coremia and sclerotia. The relative abundance of Proteobacteria in the coremia and sclerotia was 62.89 and 87.08%, respectively, followed by Bacteroidetes with a relative abundance of 34.23% in the coremia and 9.71% in the sclerotia. Firmicutes accounted for 2.28% in the coremia and 1.41% in the sclerotia. By contrast, the bacterial structure of the soil exhibited significantly different patterns. The dominant phyla in the soil samples were Acidobacteria (50.48%), Proteobacteria (29.01%), Firmicutes (10.20%), Actinobacteria (5.28%), and WPS-2 (2.80%). The fungal community of the coremia, sclerotia, and soil (Figure 3B) was dominated by Ascomycota (sclerotia, 99.97%; coremia, 97.62%, and soil, 44.84%). In addition to Ascomycota, the dominant phyla were mainly Basidiomycota (2.11%) in the coremia, and Basidiomycota (41.77%), Mucoromycota (7.80%), and Mortierellomycota (3.92%) in the soil.

It is worth noting that due to the vast number of undiscovered microorganisms and the limited resolution of the rRNA gene and the ITS region sequence, “Unclassified” made up a sizeable proportion of the OTUs at the genus level. In the bacterial community (Figure 3C), the most abundant genera detected in the coremia were *Pedobacter* (26.35%), f__Enterobacteriaceae_Unclassified (12.81%), *Pandoraea* (8.29%), *Allorhizobium–Neorhizobium–Pararhizobium–Rhizobium* (7.49%), *Phyllobacterium* (7.17%), *Chitinophaga* (7.12%), and *Stenotrophomonas* (3.63%). The most abundant genera detected in the sclerotia were *Achromobacter* (14.92%), f__Enterobacteriaceae_Unclassified (12.85%), *Stenotrophomonas* (12.51%), *Burkholderia–Caballeronia–Paraburkholderia* (11.39%), *Allorhizobium–Neorhizobium–Pararhizobium–Rhizobium* (9.19%), *Serratia* (6.11%), *Pedobacter* (5.43%), *Comamonas* (5.09%), *Pandoraea* (3.78%), f__Mitochondria_Unclassified (3.57%), and *Chitinophaga* (3.42%). In the soil samples, *Burkholderia–Caballeronia–Paraburkholderia* (5.21%), *Bacillus* (5.10%), *Acidibacter* (4.11%), f__Xanthobacteraceae_Unclassified (4.05%), and *Candidatus_Solibacter* (3.83%) were the most abundant genera. In the fungal community (Figure 3D), *Isaria* and *C. cicadae* were unsurprisingly dominant, accounting for 83.48, 53.57, and 16.50% in the sclerotia, coremia, and soil, respectively. Other major genera in soil included f__Clavariaceae_Unclassified (13.96%), *Umbelopsis* (6.18%), f__Chaetomiaceae_Unclassified (4.03%), *Mortierella* (3.80%), f__Sordariaceae_Unclassified (3.58%), and *Arcopilus* (3.42%). Other major genera in the coremia included *Apiospora* (5.55%) and *Cladosporium* (3.99%). *Fusarium* was also highly abundant in the sclerotia, reaching 15.82%.

### Interactions Between Environmental Factors and the Microbial Community

**FIGURE 4:**
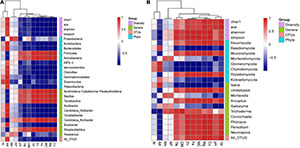
The correlation between physicochemical parameters and alpha-diversity indices, all phyla, top 10 genera, and all operational taxonomic units (OTUs) of **(A)** bacterial and **(B)** fungal communities. Red represents a positive correlation, and blue represents a negative correlation (*p < 0.05, **p < 0.01, and ***p < 0.001).

In order to elucidate the interactions between environmental factors and the soil microbial community, we assessed Pearson’s correlation between soil physicochemical parameters and all phyla, the top 10 abundant genera, all OTUs, and the alpha-diversity indices (Figure 4).

For bacteria (Figure 4A), Chao1, Shannon, and Simpson were significantly negatively correlated with Mg, while ACE was significantly negative correlated with Cu (*p* < 0.01). All bacterial OTUs were significantly negatively correlated with Mg (*p* < 0.05). The phyla Acidobacteria, Chloroflexi, and Patescibacteria were significantly negatively correlated with Cu (*p* < 0.05), while Verrucomicrobia and Patescibacteria were significantly negatively correlated with Mg (*p* < 0.05). Conversely, Firmicutes and Actinobacteria were significantly positively correlated with Mg (all *p* < 0.05 at least), and Actinobacteria were significantly positively correlated with Cu (*p* < 0.05). The dominant genera *Acidibacter*, *Candidatus Solibacter*, *Occallatibacter*, *Bryobacter*, and *Roseiarcus* were negatively correlated with most environmental factors. Among them, *Acidibacter*, *Candidatus Solibacter*, and *Bryobacter* were significantly negatively correlated with Cu (all *p* < 0.05 at least), while *Candidatus Solibacter* and *Occallatibacter* were significantly negatively correlated with Zn (all *p* < 0.05 at least). Additionally, *Burkholderia–Caballeronia–Paraburkholderia*, *Bacillus*, *Tumebacillus*, *Candidatus Koribacter*, and *Streptacidiphilus* were negatively correlated with most environmental factors. Among them, *Burkholderia–Caballeronia–Paraburkholderia*, *Bacillus*, and *Tumebacillus* were significantly positively correlated with Mg (all *p* < 0.05 at least), *Candidatus Koribacter* was significantly positively correlated with Fe and MC (*p* < 0.05), and *Streptacidiphilus* was significantly positively correlated with Cu (*p* < 0.05).

For fungi (Figure 4B), Chao1 and ACE were significantly positively correlated with TP (*p* < 0.01), while Shannon and Simpson were significantly positively correlated with Zn (*p* < 0.01) and Fe (*p* < 0.05). All fungal OTUs were significantly positively correlated with TP (*p* < 0.05). Ascomycota, the most abundant phylum, was positively correlated with TN, OM, Zn, Fe, MC, Mg, Cu, pH, and TP. By contrast, most of the other phyla presented negative correlations with most environmental factors. Among them, Basidiomycota, Chytridiomycota, and Kickxellomycotina were significantly negatively correlated with Mg, TN, and TP, respectively (all *p* < 0.05 at least). Mucoromycota and Rozellomycota were significantly positively correlated with Mg (*p* < 0.001). At the genus level, the dominant genus *Isaria* was significantly negatively correlated with Fe, MC, and Zn (all *p* < 0.05 at least). By contrast, there was a significant positive correlation between most other dominant genera and Mg (all *p* < 0.01 at least).

### Identifying the *Cordyceps cicadae* Strain

**FIGURE 5:**
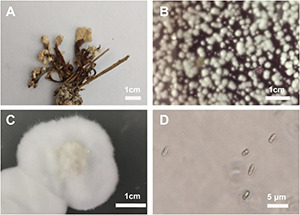
Characteristics of the *Cordyceps cicadae* strain AH10-4 isolated from the coremium. **(A)** The coremium of wild cicada flower. **(B)** Rose bengal agar (RBA) plate coated with spore suspension from the coremium. **(C)** Colony of AH10-4 cultured on potato-dextrose agar (PDA) plate. **(D)** Dark-field optical micrograph showing the spore morphology of AH10-4.

According to the rDNA sequencing results, *Isaria* was the most dominant fungal genus in all samples, including the coremia, sclerotia, and soil; and this fungus also produces the medicinally valuable fruiting bodies known in China as cicada flowers. Based on this, isolating a high yield and high activity strain of *C. cicadae* is a critical step for the large-scale artificial cultivation of this medicinal ingredient. Therefore, we isolated a fungal strain via a culture-dependent method (Figure 5). In order to identify this fungal strain, we amplified the ITS gene sequences by PCR and sequenced the obtained amplicons, and the results were aligned with sequence data in the NCBI database using the BLAST algorithm.

**FIGURE 6:**
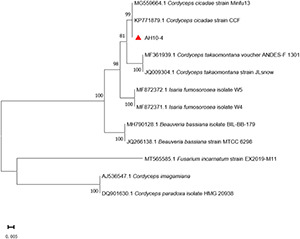
Phylogenetic tree of strain AH10-4. Evolutionary distances were assessed using the neighbor-joining method. Phylogenetic tree shows that AH10-4 belongs to the *Cordyceps cicadae*. GenBank accession numbers for ITS sequences are placed before the taxon name. *Fusarium incarnatum* were designed as the outgroup.

The phylogenetic tree was constructed using *Cordyceps*-related sequences in GenBank. The genetic distance between the isolated strain AH10-4 and *C. cicadae* strain CCF (GenBank accession: KP771879) was the closest, with the rDNA sequence identity reaching 100%. After the sequence data were combined with morphological characteristics used to determine the taxonomic status of wild strain, AH10-4 (GenBank accession: MZ676080) was identified as *C. cicadae* (Figure 6).

### Quantification of the Nucleoside Content

Nucleosides are the major bioactive constituents of *Cordyceps* and as such play important roles in the regulation and modulation of various physiological processes in the body (Meng et al., 2019). In order to test the fermentation ability of the isolated *C. cicadae* strain AH10-4, the content of nucleosides in the fermented mycelia was quantified and compared with that of the coremia and sclerotia of wild cicada flower using HPLC.

**FIGURE 7:**
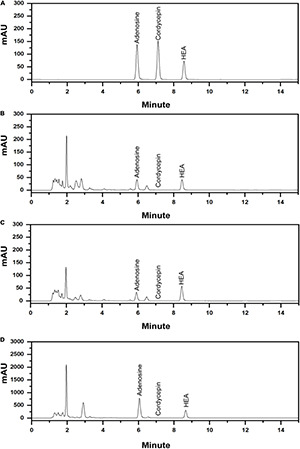
High-performance liquid chromatography (HPLC) chromatograms of **(A)** mixed standards of adenosine, cordycepin, and *N*^6^-(2-hydroxyethyl)-adenosine (HEA) and **(B)** the extract of the coremium; **(C)** the extract of the sclerotium; and **(D)** the extract of the mycelia of *C. cicadae* AH10-4.

The established analytical method was applied to simultaneously determine the content of adenosine, HEA, and cordycepin (Figure 7A), the representative nucleosides of cicada flower. For wild cicada flower, the adenosine content was 0.224 ± 0.005–0.518 ± 0.022 mg⋅g^–1^ in the coremia and 0.317 ± 0.008–0.927 ± 0.037 mg⋅g^–1^ in the sclerotia. The content of HEA was 0.268 ± 0.004–1.034 ± 0.024 mg⋅g^–1^ in the coremia and 0.190 ± 0.003–0.691 ± 0.015 mg⋅g^–1^ in the sclerotia (Figures 7B,C). The content of adenosine and HEA in the coremia and sclerotia of wild cicada flowers are shown in Table 3. Compared with that of the wild cicada flower, the average content of adenosine and HEA in the artificially cultivated mycelia increased greatly and, respectively, reached 3.042 ± 0.235 and 2.357 ± 0.207 mg⋅g^–1^ in AH10-4 mycelia (Figure 7D). However, cordycepin was not detected in any of the samples studied here.

**TABLE 3:** Contents of adenosine and HEA in different parts of wild cicada flower fruiting bodies.

**Sample**	**Position**	**Adenosine (mg⋅g^–1^)**	**HEA (mg⋅g^–1^)**
1	Coremium	0.518 ± 0.022	0.667 ± 0.014
	Sclerotium	0.927 ± 0.037	0.691 ± 0.015
2	Coremium	0.224 ± 0.005	0.268 ± 0.004
	Sclerotium	0.317 ± 0.008	0.190 ± 0.003
3	Coremium	0.284 ± 0.006	1.034 ± 0.026
	Sclerotium	0.516 ± 0.021	0.701 ± 0.022
4	Coremium	0.308 ± 0.008	0.741 ± 0.024
	Sclerotium	0.349 ± 0.011	0.327 ± 0.005
5	Coremium	0.433 ± 0.018	0.455 ± 0.009
	Sclerotium	0.601 ± 0.024	0.439 ± 0.008
6	Coremium	0.386 ± 0.014	0.611 ± 0.013
	Sclerotium	0.335 ± 0.009	0.221 ± 0.004

*HEA, N^6^-(2-hydroxyethyl)-adenosine.*

Adenosine and HEA, the most important active substances of wild cicada flower, were also found in cultivated mycelia of *C. cicadae*, and their content was significantly higher than in wild fruiting bodies (*p* < 0.05). Notably, the *C. cicadae* strain AH10-4 isolated in this study exhibited higher adenosine and HEA productivity as compared with relative researches (Chunyu et al., 2019; Ke and Lee, 2019; Qu et al., 2019), indicating that it may be a viable substitute for the rare and expensive fruiting bodies found in nature.

## Discussion

In this study, we comprehensively analyzed the physicochemical and microbial parameters in the habitat of cicada flower and the microbial structure of its fruiting bodies in Anji County, Zhejiang Province.

Previous studies have shown that soil moisture affects microbial activity, gene expression, and community composition (Gartzia-Bengoetxea et al., 2016), and the requirement of *Cordyceps* for specific ranges of MC and soil acidity–alkalinity in soil layers is very high (He et al., 2017). Our soil analysis indicated that cicada flower preferentially occurs in acidic soil (pH 5.9) with abundant MC, TN, and OM, which was consistent with previous reports that the soil around Chinese *Cordyceps* was also acidic (pH 5.37–5.41) and that the MC was ∼40% (Shao et al., 2019). Mushrooms grown in nature tend to assimilate including metals (Ko et al., 2017). As we all know, Zn has physiological and nutritional functions in traditional Chinese medicine and can regulate enzyme activity; Fe is a cofactor of many enzymes, such as cytochromes and peroxidases; and Cu plays an important role in cell wall metabolism and mitochondrial respiration; they are all essential microelements for plants. Our correlation analysis between physicochemical properties and microbial community of soil demonstrated that Zn, Fe, and MC were significantly negatively correlated with the presence of the most dominant fungal genus *Isaria*, and Cu was significantly negatively correlated with most dominant soil bacterial genera. But Mg was significantly positively correlated with most dominant taxa. It should be noted that the results (Figure 4) of this study indicated that *Isaria* is significantly negatively correlated with MC, TN, TP, and OM, but our results of soil physicochemical properties (Table 1) showed that cicada flower grows in high-humidity soil, and it should only occur when the soil moisture reached a certain level. However, increasing the water content above the optimal level might inhibit the growth of *Isaria* and thus inhibit the occurrence of its medicinally valuable fruiting bodies, which implicated that in the artificial cultivation of cicada flower, various soil physicochemical factors must be controlled within a reasonable range, so as to promote the occurrence of cicada flower. Soil is a rather complicated matrix, and all of the physicochemical factors might also affect cicada larvae, whereby the occurrence of cicada flower might be the outcome of the synergy of these factors.

By analyzing the fungal and bacterial communities inhabiting cicada flower and the surrounding soil microenvironment, we revealed that soil had the highest abundance of both fungal and bacterial species (Table 2), which was consistent with previous studies (Brodie et al., 2003; Tilman et al., 2006; Sheik et al., 2011; Meyer et al., 2014). However, the diversity of fungi in the coremia and sclerotia was lower, especially the lowest in the sclerotia, which indicated that the diversity of fungi may not be conducive to the occurrence of cicada flower because of its possible robust competition for survival to other fungi during formation of fruiting body. The microbial composition (Figure 2) of the soil presented evident differences with that of the coremia and sclerotia, because the disclosure of external microbial environment of cicada flower was richer and more diverse than that of the internal microbial environment (Table 2), suggesting that the potent *C. cicadae* might inhibit some microbes unable to colonize during infection. The microbial composition (Figure 2) in the coremia and sclerotia presented evident differences with that of the soil environment. Thus, the colonizing microbial community may reshape the internal environment. This speculation can be proved in a subsequent analysis of the microbial community of cicada larvae. The differences of microbial composition in the coremia and sclerotia are not distinct, indicating that during the formation of the coremia, some of the microorganisms colonizing the sclerotia might be transported into the coremia. The external microbial environment of cicada flower was richer and more diverse than the internal microbial environment, suggesting that *C. cicadae* may have inhibited some microbes unable to colonize during infection.

In terms of the composition of microbial taxa, unsurprisingly, *Isaria* was the most dominant fungal genus and *C. cicadae* was the dominant in the internal and external microbial communities of wild cicada flower, accounting for 83.48, 53.57, and 16.50% in the coremia, sclerotia, and soil, respectively, indicating that the genetics of the species *C. cicadae* was relatively stable; and the formation of cicada flower was mainly caused by this fungus (Chunyu et al., 2019), and the formation mechanism of cicada flowers is probably the same as that of other species of *Cordyceps*. The fungal spores erupt from the coremia of cicada flower randomly scatter in the top soil, gradually infiltrate into deeper soil layers with rainwater, develop into infective conidia that secrete chitinase, and enter the larvae of cicada (Shao et al., 2019). Compared with that of *Isaria*, the relative abundance of other fungal genera was very low, indicating the strong competition between *C. cicadae* and other fungi. Similar to previously published research, that *C. cicadae* injects into the pupae may inhibit some bacterial genera (Zeng et al., 2021). Among the bacteria, the dominant genera *Allorhizobium–Neorhizobium–Pararhizobium–Rhizobium*, Enterobacteriaceae, *Stenotrophomonas*, *Pandoraea*, and *Chitinophaga* attracted our attention. These genera occupied more than 29 and 50% in the coremia and sclerotia, respectively, and less than 1% in soil. *Allorhizobium–Neorhizobium–Pararhizobium–Rhizobium*, belonging to non-cyanobacteria diazotrophic genera that are known to be associated with plant roots (You et al., 2021), was the dominant genus in the sclerotia and coremia, which might support the previous speculation that cicada flower may exchange carbon and nitrogen elements in soil via endophytic bacteria to affect the content of carbon and nitrogen compounds in cicada flower (Zeng et al., 2021). A previous study found that four strains of Enterobacteriaceae isolated from wild cicada flower can inhibit *C. cicadae* on PDA medium and increase the yield of fungal metabolites of *C. cicadae* (Qu et al., 2019), and Enterobacteriaceae may play an important role in host fitness by resisting pathogenic microbes (Hu et al., 2020), so we speculated that Enterobacteriaceae might have an inhibitory effect on *C. cicadae* and may help *C. cicadae* to resist other pathogens. In the process of infecting the host, *C. cicadae* needs chitinases to dissolve the insect epidermis, and the main ability of *Chitinophaga* is to degrade chitin (Sharma et al., 2020). Therefore, we speculated that *Chitinophaga* might facilitate *C. cicadae* to infect cicada nymphs and larvae; after all, some members of this genus also were found to solubilize phosphate and zinc compounds, assisting the utilization of nutrients by plants, contributing to enhanced soil fertility, and promoting plant growth (Chung et al., 2012). In the growth process of *C. cicadae*, it can interact with other microbes to obtain basic nutrients like carbon sources, and *Stenotrophomonas* is able to digest carboxymethylcellulose (Dantur et al., 2015). *Pandoraea* could be involved in oxalate degradation, which may be important contributors to soil formation, soil fertility and retention, and cycling of elements necessary for plant growth (Peeters et al., 2019). We therefore speculated that these bacteria might provide nutrition for *C. cicadae* during the growth of cicada flowers.

Additionally, higher quality and yield of the fermentation products and shortening the culture time have become a crucial topic for the development of cicada flower. The *C. cicadae* strain AH10-4, which we isolated here, is a highly productive strain with excellent production of adenosine and HEA; it can be further genetically modified and optimized for artificial cicada flower cultivation in the future.

We believe that by systematically exploring the internal and external physicochemical characteristics and microbial community of cicada flower, an emphasis on their influence on its occurrence will assist in the future discovery of undetected potential microbial bio-resources, which can be used to develop alternatives to the unsustainable harvesting of naturally occurring wild cicada flower.

## Data Availability Statement

The datasets presented in this study can be found in online repositories. The names of the repository/repositories and accession number(s) can be found below: https://www.ncbi.nlm.nih.gov/, PRJNA751148.

## Author Contributions

HR, WS, and SW conceived and designed the experiments. AH, TW, and BZ drafted the manuscript. HR and WS collected the samples. AH and XW performed the experiments. AH, BZ, and YS collected and analyzed the data. HR, WS, and TW participated in the study design, technological guidance, and coordination. All authors contributed to the article and approved the submitted version.

## Conflict of Interest

The authors declare that the research was conducted in the absence of any commercial or financial relationships that could be construed as a potential conflict of interest.

## Publisher’s Note

All claims expressed in this article are solely those of the authors and do not necessarily represent those of their affiliated organizations, or those of the publisher, the editors and the reviewers. Any product that may be evaluated in this article, or claim that may be made by its manufacturer, is not guaranteed or endorsed by the publisher.

## Footnotes

1
https://unite.ut.ee/
