# Supplementary material for: Analysis of Internal and External Microorganism Community of Wild Cicada Flowers and Identification of the Predominant Cordyceps cicadae Fungus
Source: Front Microbiol. 2021 Nov 25;12:752791. doi: 10.3389/fmicb.2021.752791 (PMC8656164; doi:10.3389/fmicb.2021.752791)
Supplement: Supplementary file 1 [file Data_Sheet_1.zip › AdditionalFiles-Revised/Supplementary Material Presentation/Supplementary Materials/Caption for supplementary data.docx]

**Caption for supplementary data**

**1. Table**

(1) Table S1. The shared and unique bacterial OTUs among the soil, coremia and sclerotia samples.

(2) Table S2. The shared and unique fungal OTUs among the soil, coremia and sclerotia samples.

(3) Table S3. The detailed taxonomic information of the bacterial OTUs.

(4) Table S4. The detailed taxonomic information of the fungal OTUs.

**2. Figure**

(1)


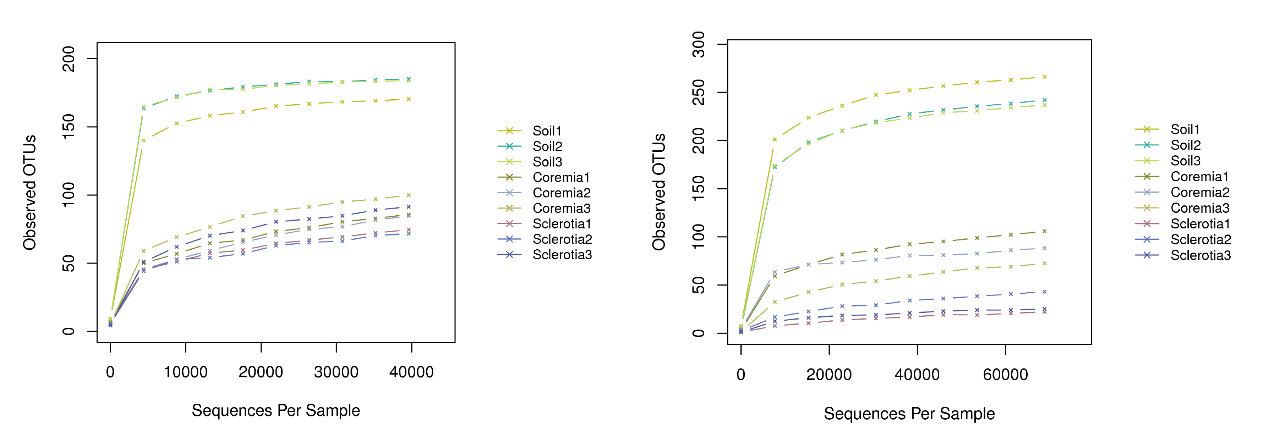


1. (B)

Figure S1 Rrarefaction curves of (A) bacterial and (B) fungal communities in the samples of soil, coremia, and sclerotia.

(2)


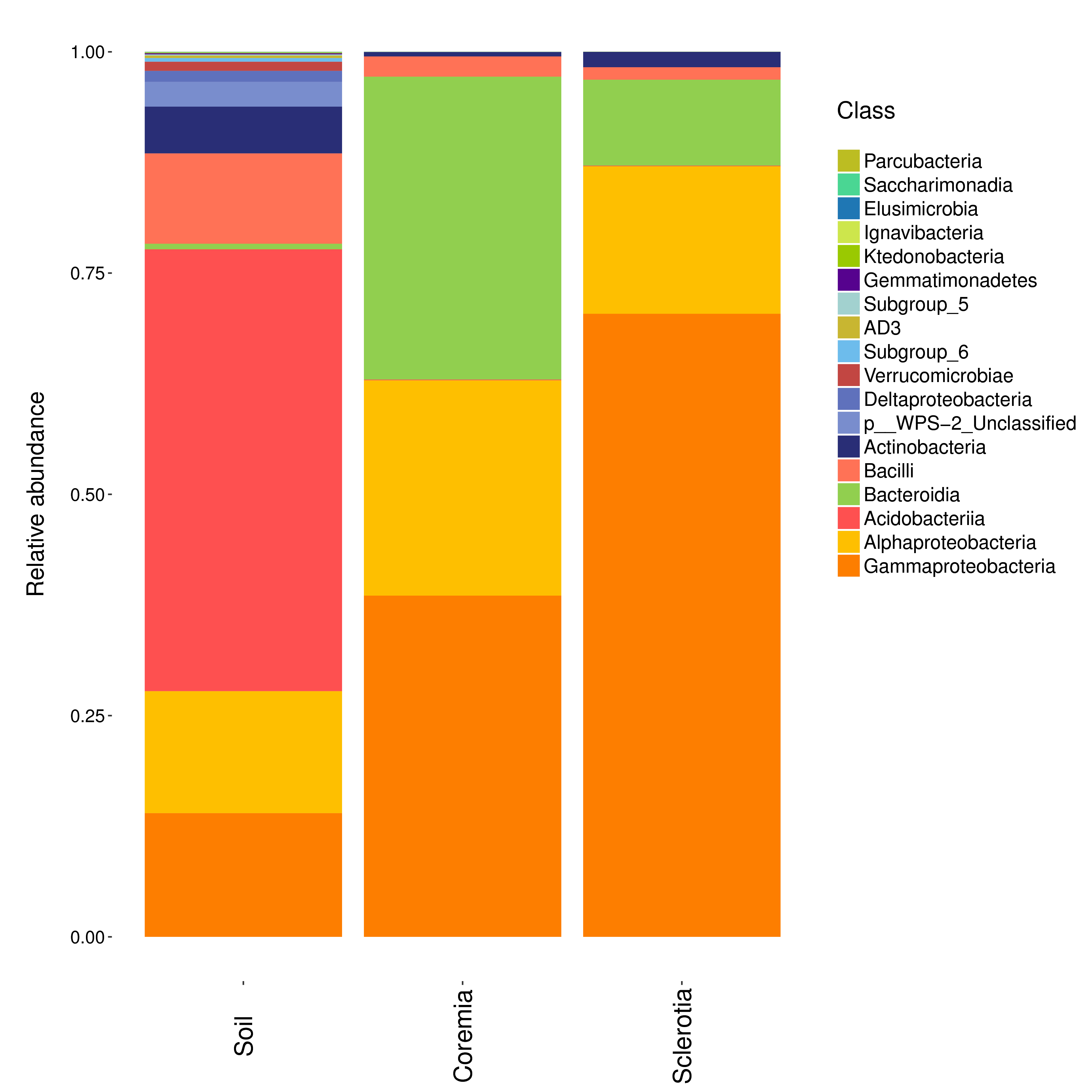


Figure S2 Structure of the endogenous bacterial community of cicada flower and its microhabitat samples at the class level.

(3)


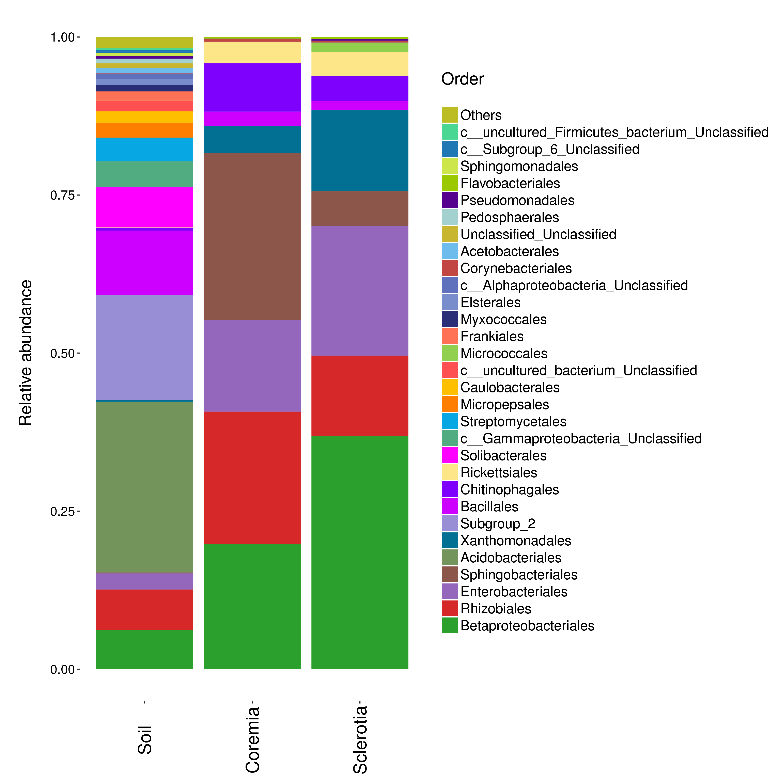


Figure S3 Structure of the endogenous bacterial community of cicada flower and its microhabitat samples at the order level.

(4)


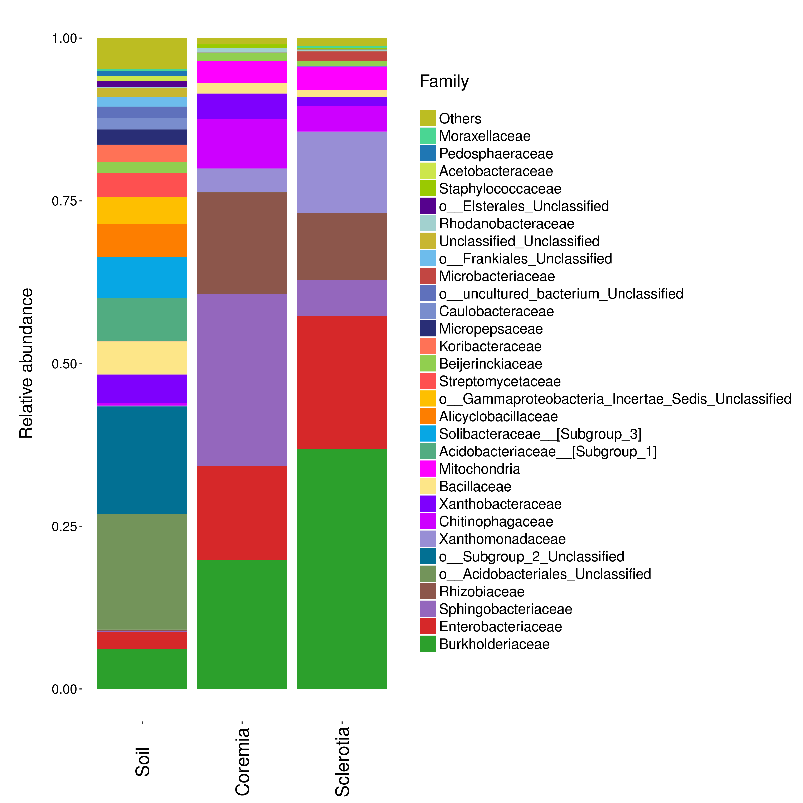


Figure S4 Structure of the endogenous bacterial community of cicada flower and its microhabitat samples at the family level.

(5)


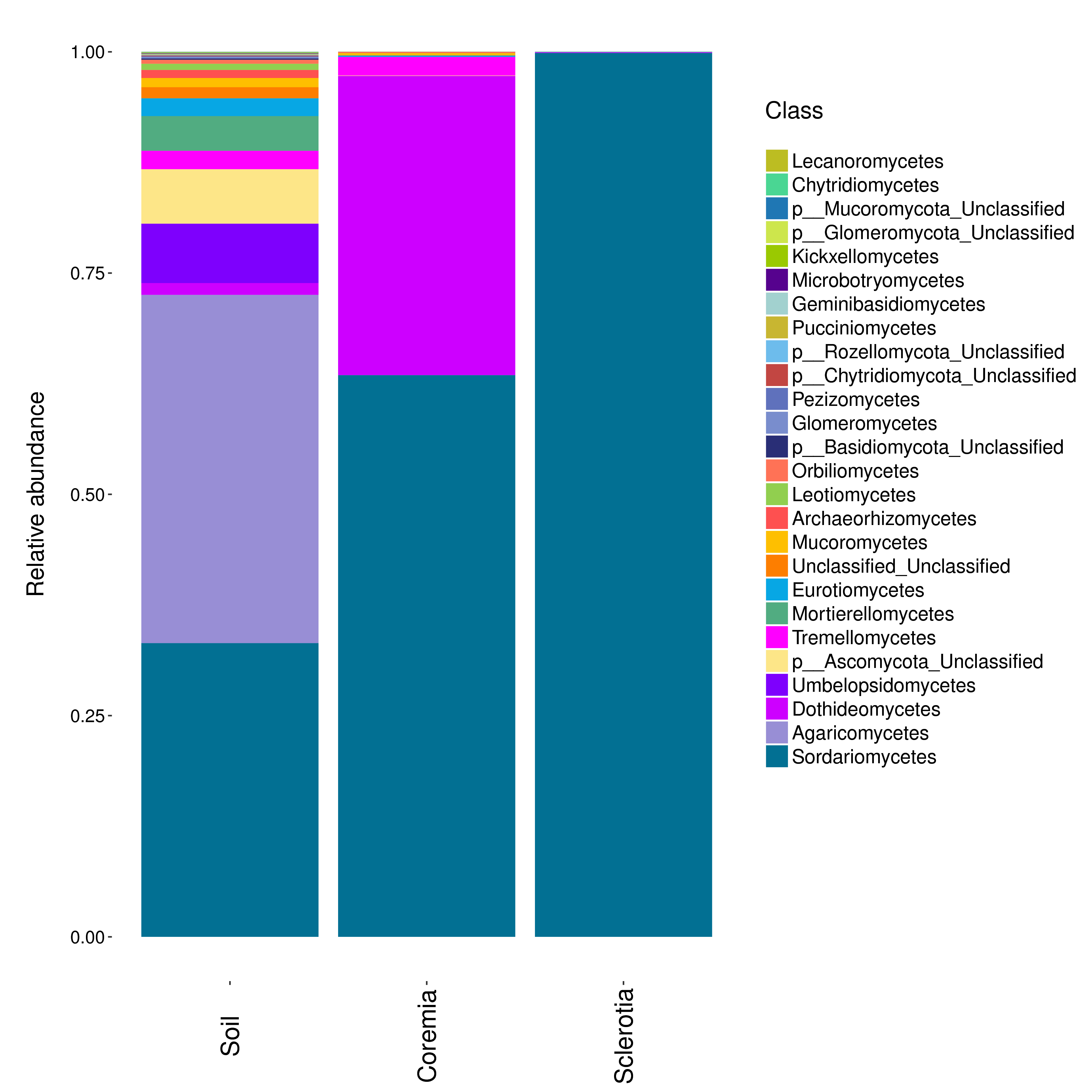


Figure S5 Structure of the endogenous fungal community of cicada flower and its microhabitat samples at the class level.

(6)


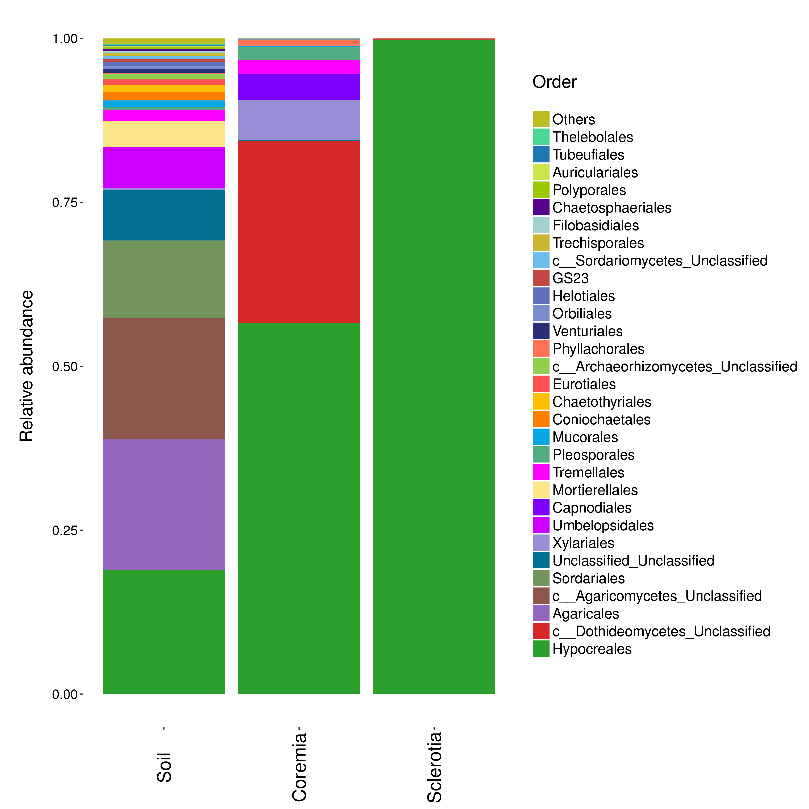


Figure S6 Structure of the endogenous fungal community of cicada flower and its microhabitat samples at the order level.

(7)


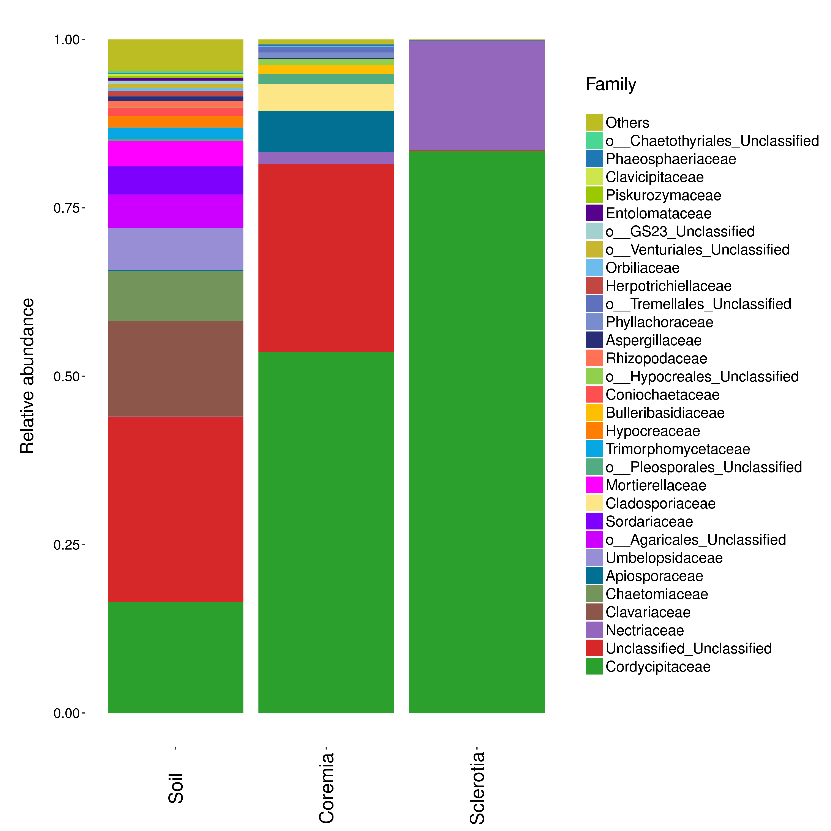


Figure S7 Structure of the endogenous fungal community of cicada flower and its microhabitat samples at the family level.
